# Quantitative assessment of diffuse myocardial fibrosis and edema in patients with and without cardiac involvement of sarcoidosis by cardiovascular magnetic resonance T1- and T2-mapping at 1.5T

**DOI:** 10.1186/1532-429X-18-S1-P255

**Published:** 2016-01-27

**Authors:** Darius Dabir, Julian A Luetkens, Daniel Kuetting, Rami Homsi, Christian Marx, Folke Kluenker, Hans H Schild, Daniel K Thomas

**Affiliations:** grid.10388.320000000122403300Department of Radiology, University of Bonn, Bonn, Germany

## Background

Cardiac involvement of sarcoidosis hallmarked by inflammatory myocardial processes and subsequent fibrotic myocardial alterations is a life threatening condition that makes early diagnosis favorable. Native T1- as well as T2-mapping have been proposed as reliable methods to non-invasively assess diffuse myocardial fibrosis and edema respectively. Our aim was to examine the value of both methods for the differentiation between healthy and diseased myocardium.

## Methods

Patients with sarcoidosis who previously underwent a CMR examination at our hospital were invited for a follow up scan. The follow up scan performed on a 1.5T Philips INGENIA scanner comprised a clinically routine CMR-protocoll with additional T1- and T2-mapping in midventricular short axis (SA) slice. T1-mapping was performed using MOdified Look Locker Inversion Recovery Imaging (3-3-5 MOLLI) and T1-relaxation times were quantified within the septal myocardium (CONSEPT-approach). For T2 mapping an optimized 6-echo gradient spin echo (GraSE) sequence was used measuring T2 relaxation times within the whole SA slice. CMR exams were classified as "cardiac involvement" if at least one of the following pathologies occured: pathological relative enhancement, late enhancement findings consistent with (post-) inflammatory changes, myocardial edema, pericardial effusion. According to CMR results patients were divided into 4 different groups: group 1 (n = 10): patients with positive findings in both exams, group 2 (n = 6): patients with positive findings only in the initial exam; group 3 (n = 8): patients with sarcoidosis but inconspicuous initial CMR exam and group 4 (n = 20): a control group of healthy age matched volunteers with no medical history of sarcoidosis or cardiac disease.

## Results

Results are shown in Table 1.

**Table 1:** Table 1

	Age (years)	LVEDV (ml)	IVSD (mm)	EF (%)	T1 (ms)	T2 (ms)
Group 1 (n=10)	51,80 ± 28,28	134,55 ± 42,42	9,11 ± 0,71	58,78 ± 7,07	1009,30 ± 13,44 (p <0,05)	54 ± 1,41
Group 2 (n=6)	54,33 ± 5,66	117 ± 21,92	9,20 ± 1,06	60,8 ± 18,38	988 ± 10,61	54,17 ± 2,12
Group 3 (n=8)	55,13 ± 11,31	110,57 ± 16,26	9,69 ± 0,71	66,50 ± 1,41	1001,86 ± 0,71	54,50 ± 2,08
Group 4 (n=20)	50,60 ± 31,82	150,40 ± 61,52	9,96 ± 0,71	62,55 ± 4,24	957,65 ± 15,56	52,95 ± 4,95

Patient characteristics as well as T1 and T2 relaxation times.

T1 relaxation times were significantly longer in patients with positive CMR findings in both the initial examination and the follow-up scan (group 1) in comparison to all other groups (p < 0,05). However no significant difference in T1 relaxation time could be revealed in patients with history of cardiac involvement of sarcoidosis (group 2) as well as patients with sarcoidosis but no CMR findings (group 3; p < 0,05). No significant difference in T2 relaxation times could be revealed between all 4 groups (p < 0,05).

## Conclusions

Patients with consistent cardiac involvement of sarcoidosis have significantly increased T1 relaxation times as a sign of diffuse cardiac fibrosis whereas no relation between diffuse myocardial edema and cardiac involvement of sarcoidosis could be revealed.

